# Exercise intensity–dependent metabolic adaptations in streptozotocin-induced diabetic rats: modulatory effects of apple cider vinegar on inflammatory and endocrine biomarkers

**DOI:** 10.3389/fphys.2026.1842179

**Published:** 2026-09-03

**Authors:** Mehmet Kaan Akay, Zarife Pancar, Muhammed Kaan Darendeli, Mustafa Sencer Ulema, Burak Karaca, Hasan Ulusal, Ahmet Sarper Bozkurt, Ali Muhittin Taşdoğan, Xu Yan

**Affiliations:** 1 Department of Physical Education and Sports, Gaziantep University, Gaziantep, Türkiye; 2 Department of Physical Education and Sports, Faculty of Sports Science, Gaziantep University, Gaziantep, Türkiye; 3 Department of Medical Biochemistry, Faculty of Medicine, Gaziantep Islam Science and Technology University, Gaziantep, Türkiye; 4 Department of Basic Medical Sciences, Faculty of Medicine, Gaziantep University, Gaziantep, Türkiye; 5 Department of Anesthesiology and Reanimation, Faculty of Medicine, Gaziantep University, Gaziantep, Türkiye; 6 Institute for Health and Sport, Victoria University, Melbourne, VIC, Australia; 7 Department of Medicine-Western Health, The University of Melbourne, Melbourne, VIC, Australia

**Keywords:** combined exercise, exercise intensity, immune system, metabolic regulation, myokine secretion

## Abstract

**Background:**

Diabetes mellitus is characterized by metabolic dysregulation and chronic inflammation, leading to impaired hormonal and biochemical homeostasis. Both exercise and nutritional interventions, such as apple cider vinegar (ACV), have been suggested as potential strategies to improve metabolic and inflammatory responses in diabetic conditions. However, the combined effects of ACV and different exercise intensities remain unclear.

**Objective:**

The present study aimed to evaluate the effects of apple cider vinegar supplementation, alone and in combination with different exercise intensities, on metabolic and inflammatory biomarkers, as well as adiponectin, betatrophin, and irisin levels in streptozotocin-induced diabetic rats.

**Methods:**

A total of 80 rats were randomly assigned into 10 groups (n = 8 per group), including control, sham, diabetes, diabetes+supplementation, diabetes+low-, moderate-, and high-intensity treadmill exercise, and diabetes+supplementation combined with low-, moderate-, and high-intensity exercise. Diabetes was induced using streptozotocin, and ACV was administered via oral gavage. Following a one-week adaptation period, exercise groups performed treadmill training at different intensities for 4 weeks. Following euthanasia by decapitation, blood samples were collected from the jugular vein and analyzed using ELISA. Statistical analyses were conducted using paired samples t-tests for within-group comparisons and one-way ANOVA with Tukey’s *post hoc* test for between-group comparisons.

**Results:**

Diabetic rats showed significant impairments in adiponectin, betatrophin, irisin, IL-1β, IL-6, TNF-α, AST, ALT, and insulin levels compared to controls. ACV supplementation alone significantly improved several biomarkers, including betatrophin, IL-6, TNF-α, AST, ALT, and insulin. Exercise interventions also led to significant improvements, with low- and moderate-intensity exercise demonstrating greater benefits than high-intensity exercise. Moreover, the combination of ACV and low-intensity exercise was associated with the most favorable biomarker profile across multiple metabolic and inflammatory markers.

**Conclusion:**

In this STZ-induced rat model, ACV supplementation and low-to-moderate intensity exercise modulate metabolic and inflammatory biomarkers more effectively than higher-intensity protocols. Whether these findings translate to clinical settings requires confirmation in human studies; no synergistic effects or therapeutic recommendations can be concluded without formal interaction testing and clinical validation.

## Introduction

Diabetes mellitus (DM) is a chronic metabolic disorder characterized by persistent hyperglycemia, resulting from defects in insulin secretion, action, or both. This condition has reached pandemic levels globally, with an estimated 463 million adults living with diabetes in 2019, a figure projected to rise to 700 million by 2045 (International Diabetes Federation, 2019). DM is associated with a myriad of complications, including cardiovascular diseases, neuropathy, nephropathy, and retinopathy, which impose a substantial burden on healthcare systems and negatively affect patients’ quality of life (Zheng et al., 2018).

The management of diabetes often involves pharmacological interventions, but these may have limitations, including side effects, limited accessibility, and financial constraints. Consequently, there has been growing interest in complementary and alternative approaches, such as dietary modifications and physical activity, to manage glycemic control and reduce diabetes-associated complications (Gao et al., 2024). Among these, natural products and functional foods have attracted significant attention due to their potential therapeutic properties. Apple cider vinegar (ACV), a widely available and inexpensive natural product, has been proposed as a complementary intervention in diabetes management owing to its bioactive components, including acetic acid, phenolic compounds, and flavonoids (Morgan and Mosawy, 2016).

Several studies have demonstrated that ACV may exert beneficial effects on glycemic control, lipid metabolism, and inflammation by modulating key metabolic pathways (Hadi et al., 2021; Xia et al., 2023). Additionally, regular physical activity is well-recognized for its role in improving insulin sensitivity, reducing systemic inflammation, and enhancing overall metabolic health (Małkowska, 2024). While both ACV supplementation and exercise have been independently shown to improve diabetes-related biomarkers, the combined effects of these interventions remain underexplored, particularly in the context of varying exercise intensities.

The present study aims to address this gap by investigating the combined effects of ACV supplementation and treadmill exercise at different intensities on metabolic and inflammatory biomarkers, as well as adipokines such as adiponectin, betatrophin, and irisin, in a rat model of streptozotocin-induced diabetes. Among the biomarkers evaluated in this study, adiponectin, irisin, and betatrophin were selected on the basis of their established roles in diabetic pathophysiology and their known responsiveness to both exercise and nutritional interventions. Adiponectin, an adipose-derived hormone, is markedly reduced in diabetic states and exerts insulin-sensitizing and anti-inflammatory effects primarily through AdipoR1/R2-mediated activation of the AMPK–PPARα pathway; its circulating levels have been consistently shown to respond to exercise in an intensity-dependent manner (Guan et al., 2023; Abdalla, 2024). Irisin, a myokine generated through proteolytic cleavage of FNDC5 during skeletal muscle contraction via the PGC-1α pathway, promotes glucose uptake, mitochondrial biogenesis, and browning of white adipose tissue; its secretion is impaired in STZ-induced diabetic models, where oxidative stress suppresses FNDC5 expression (Behera et al., 2022; Guo et al., 2024). Betatrophin (ANGPTL8), expressed predominantly in the liver and adipose tissue, regulates lipoprotein lipase activity and lipid metabolism, and its circulating levels are elevated in insulin-resistant states, positioning it as a compensatory hepatokine in metabolic dysregulation (Guo et al., 2022). Collectively, these three biomarkers represent distinct but mechanistically complementary axes, endocrine (adiponectin), myokine (irisin), and hepatokine (betatrophin), whose simultaneous assessment provides a comprehensive picture of metabolic adaptation under combined dietary and exercise stress.

By elucidating the interaction between ACV and exercise, this study seeks to provide novel insights into integrative approaches for diabetes management and highlight the importance of tailoring exercise intensity to optimize therapeutic outcomes. To our knowledge, this is the first study to simultaneously examine the effects of ACV supplementation combined with three distinct exercise intensities on adiponectin, betatrophin, and irisin in a STZ-induced type 1 diabetes model, thereby addressing a critical gap in the understanding of how intensity-dependent exercise interacts with dietary supplementation at the hormonal and inflammatory level. This investigation is particularly significant given the increasing prevalence of diabetes and the urgent need for cost-effective, accessible, and evidence-based interventions. Furthermore, understanding the interplay between dietary supplementation and exercise can contribute to the development of personalized strategies for diabetes care. The findings of this study may pave the way for future clinical research and practical applications in diabetes prevention and management.

## Materials and methods

### Experimental design

A total of 80 male Wistar Albino rats, aged 8–10 weeks, were used in the study. The rats were randomly assigned to ten different groups, with eight rats in each group. Randomization was performed using a simple random assignment procedure; prior to group allocation, animals were stratified by body weight to ensure baseline comparability across groups, and assignment was carried out by an independent researcher using a numbered lottery draw. One rat in the diabetes group died during the final week of the experimental period due to severe metabolic complications associated with STZ-induced diabetes, prior to blood collection, and was therefore excluded from biochemical analyses, resulting in a final sample size of n=7 for this group. Power analysis (G*Power 3.1.9.4 version) was conducted to ensure an adequate sample size for statistical generalization and group determination (Ousaaid et al., 2020).

**Figure 1:**
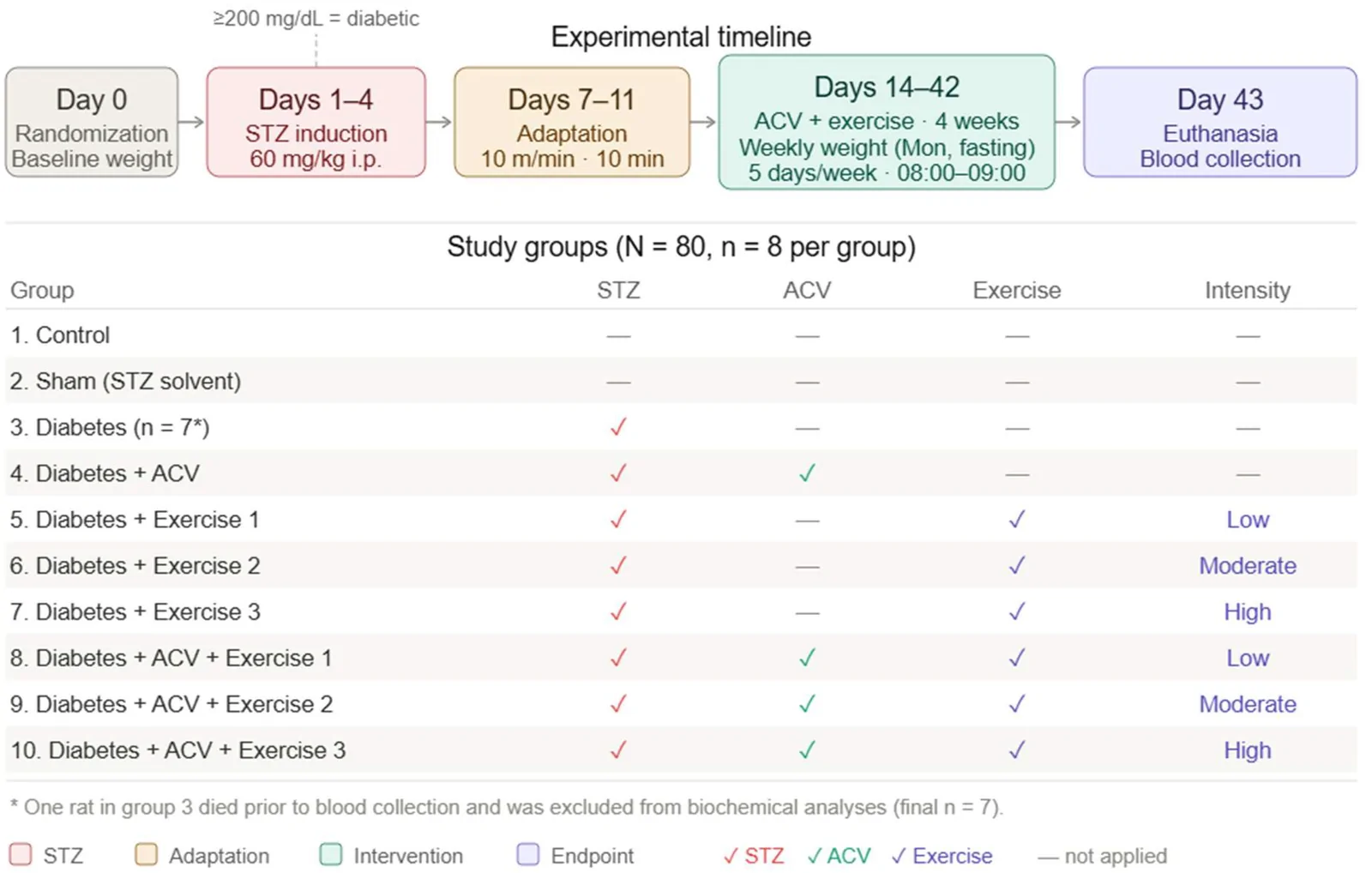
Experimental design.

The animals used in this study were obtained from the Gaziantep University Experimental Animal Research and Application Center (GAUNDAM), where all experimental procedures were carried out. Ethical approval for the study was obtained from the Gaziantep University Local Ethics Committee for Animal Experiments (approval no. 2023/21; protocol no. 314). This study was reported in accordance with the ARRIVE (Animal Research: Reporting of *In Vivo* Experiments) 2.0 guidelines to ensure transparency and reproducibility of animal research. All procedures adhered to the National Institutes of Health (NIH) guidelines for the care and use of laboratory animals. The rats were housed under controlled conditions, maintained at a temperature of 22± 3 °C, with 45% ± 5% humidity, and a 12-hour light and 12-hour dark cycle. They were kept in standard cages and fed a standard rat diet ad libitum (Arden Yem Sanayi Araştırma Deney Ticaret Ltd. Şti., Rat 3000 Yemi; crude protein 24%, metabolizable energy 3000 kcal/kg, 12 mm pellet, enriched with vitamin and mineral mixture). No dietary modifications were applied between groups; ACV gavage was administered in addition to standard daily food intake. The age and strain of the rats were selected based on previous studies involving apple cider vinegar supplementation (Ousaaid et al., 2020; Halima et al., 2018) and exercise protocols (Zeng et al., 2007; Heo et al., 2021; Lin et al., 2024). The experimental procedure is illustrated in **Figure 1**. Ethical considerations for this procedure were reviewed and approved as part of the ethical protocol, ensuring that all experimental interventions were conducted in compliance with established animal welfare standards.

The experimental timeline spanned 43 days. On Day 0, rats were randomized and baseline body weights were recorded under fasting conditions. STZ was administered intraperitoneally on Day 1, and blood glucose levels were confirmed on Day 4 (≥200 mg/dL = diabetic). Exercise adaptation commenced on Day 7 (10 m/min, 10 min/day, 5 days). The four-week intervention period (Days 14–42) included ACV administration (2 mL/kg/day, oral gavage) and exercise protocols. Body weights were recorded weekly on Monday mornings under fasting conditions. All animals were euthanized on Day 43 and blood samples were collected from the jugular vein.

### Weight monitoring

Prior to the commencement of the *in vivo* phase of the experiment (Day 0), the body weights of all experimental animals were measured and recorded after randomization into groups. Additionally, all rats were weighed at the beginning of each week (on Mondays) prior to the administration of supplementation and exercise interventions. Body weights were measured using a DESIS precision scale (ETS Elektronik Tartı Sis. San. ve Tic. Ltd. Şti., Istanbul) available at the GAUNDAM facility, and weekly weight changes were documented throughout the study period. The scale has a resolution of 0.1 g and was calibrated against certified reference weights prior to each measurement session in accordance with the manufacturer’s guidelines. For each measurement, animals were individually transferred to a tared plastic weighing container to ensure consistent handling conditions across all sessions. All measurements were taken in the morning under fasting conditions, prior to supplementation and exercise interventions.

### Apple cider vinegar supplementation

The ACV used in this study was produced through a two-stage fermentation process (alcoholic and acetic acid fermentation) using apples harvested from Pozantı, Adana, Türkiye. The bioactive composition of the ACV was verified prior to the experiment by GC-MS headspace analysis conducted at the Uluğ Bey Technology Application and Research Center (ULUTEM), Gaziantep University (Test Report No: R24-0028, dated 30.04.2024). Analysis confirmed that acetic acid was the predominant component (88.18%), accompanied by isoamyl acetate (2.96%), 2,3-butanediol (2.34%), and 1-hexanol (1.51%), among others. The physicochemical properties of the ACV were as follows: pH 3.301, density 1.031 g/cm³, and acidity 1.022 g/100 mL. The administration of ACV was based on a review of the relevant literature to establish the appropriate dosage and delivery method. Previous studies have shown that ACV is typically administered orally. To standardise the dosage and ensure that the volume administered did not exceed the maximum tolerable volume for gavage, all rats in the supplementation groups received ACV via oral gavage at a total volume not exceeding 1 mL (Ousaaid et al., 2020). The ACV was stored in a 4 °C refrigerator until the experimental procedures commenced. For the groups receiving ACV, the supplementation was provided at a dosage of 2 mL/kg/day, administered orally via gavage each morning (08:00–09:00) for five days a week over the course of four weeks (Hyun and Jang, 2016; Ousaaid et al., 2020; Bouderbala et al., 2015, 2016).

### Diabetes induction and streptozotocin administration

**Figure 2:**
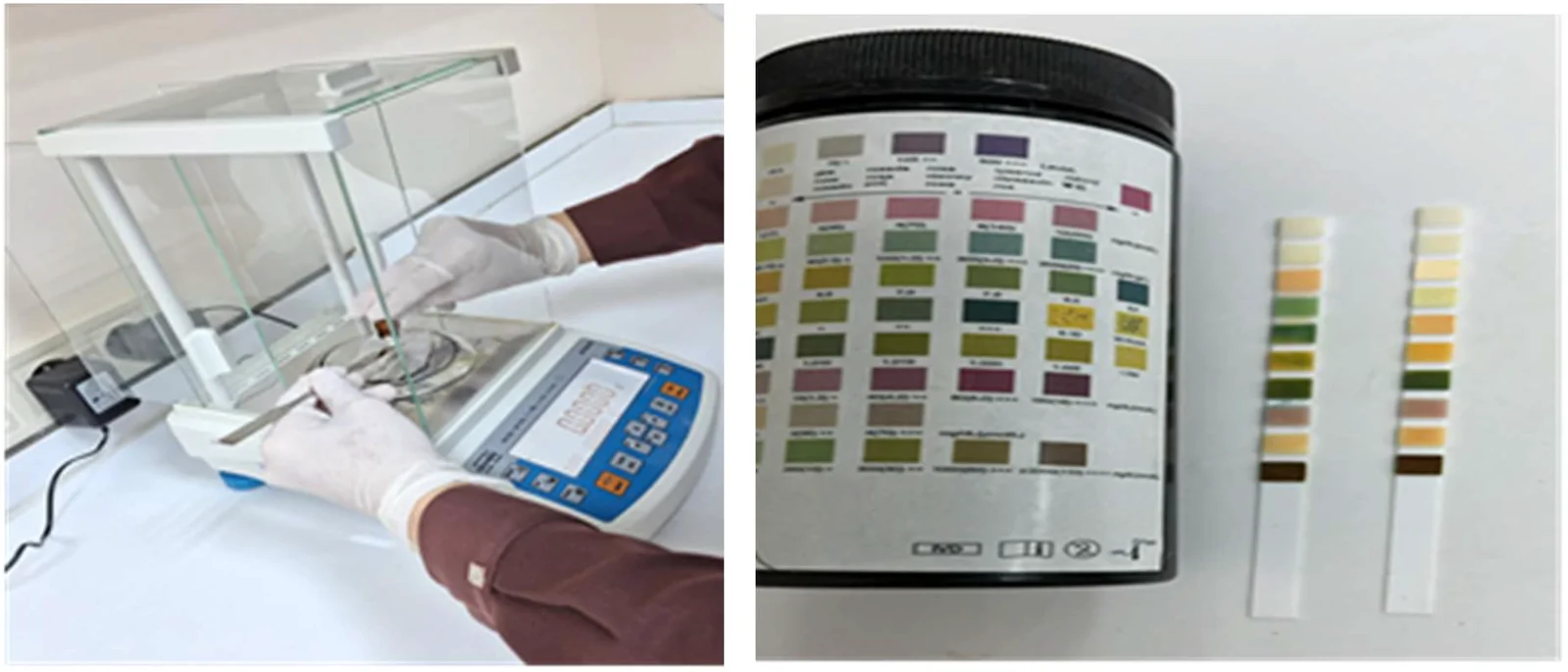
Left: during the preparation of STZ, the powdered compound was carefully measured using a precision balance to ensure accurate dosing. Right: urine test strip results. The urine analysis strips revealed elevated glucose levels, indicating a diabetic condition. The strip results, shown in the figure, confirm increased urinary glucose concentrations associated with uncontrolled diabetes. These results were interpreted using the color scale as a reference.

Streptozotocin (STZ) is widely used to induce type 1 diabetes in animal models and also leads to chronic endogenous stress (Damasceno et al., 2014). STZ-induced diabetes is characterised by significant structural and functional abnormalities in both peripheral and central nerve fibers (Muramatsu et al., 2017; Voitenko et al., 2000). In this study, diabetes was induced in rats by dissolving STZ in 0.1 M phosphate-citrate buffer (pH 4.5) and administering it intraperitoneally (i.p.) at a single dose of 60 mg/kg. The STZ solution was freshly prepared before each injection to ensure stability and accurate dosing. Specifically, 10 mg of STZ was dissolved in 200 mL of citrate buffer (0.1 M, pH 4.5), and the solution was stored on ice and used within 2 hours (**Figure 2**).

Seventy-two hours after STZ injection, blood glucose levels were measured using a glucometer (Accu-Chek Active, Roche Diagnostics, Germany). Blood samples were collected from the tail vein using a minimally invasive technique to minimise stress on the animals. Rats with blood glucose levels exceeding 200 mg/dL were considered diabetic (Xie et al., 2020; Huang et al., 2020). Monitoring and handling protocols were strictly followed during and after the procedure to ensure the welfare of animals. Additionally, glucose levels were also monitored in the rats’ urine to minimise stress caused by repeated blood sampling from the tail vein. .

### Exercise protocols

**Figure 3:**
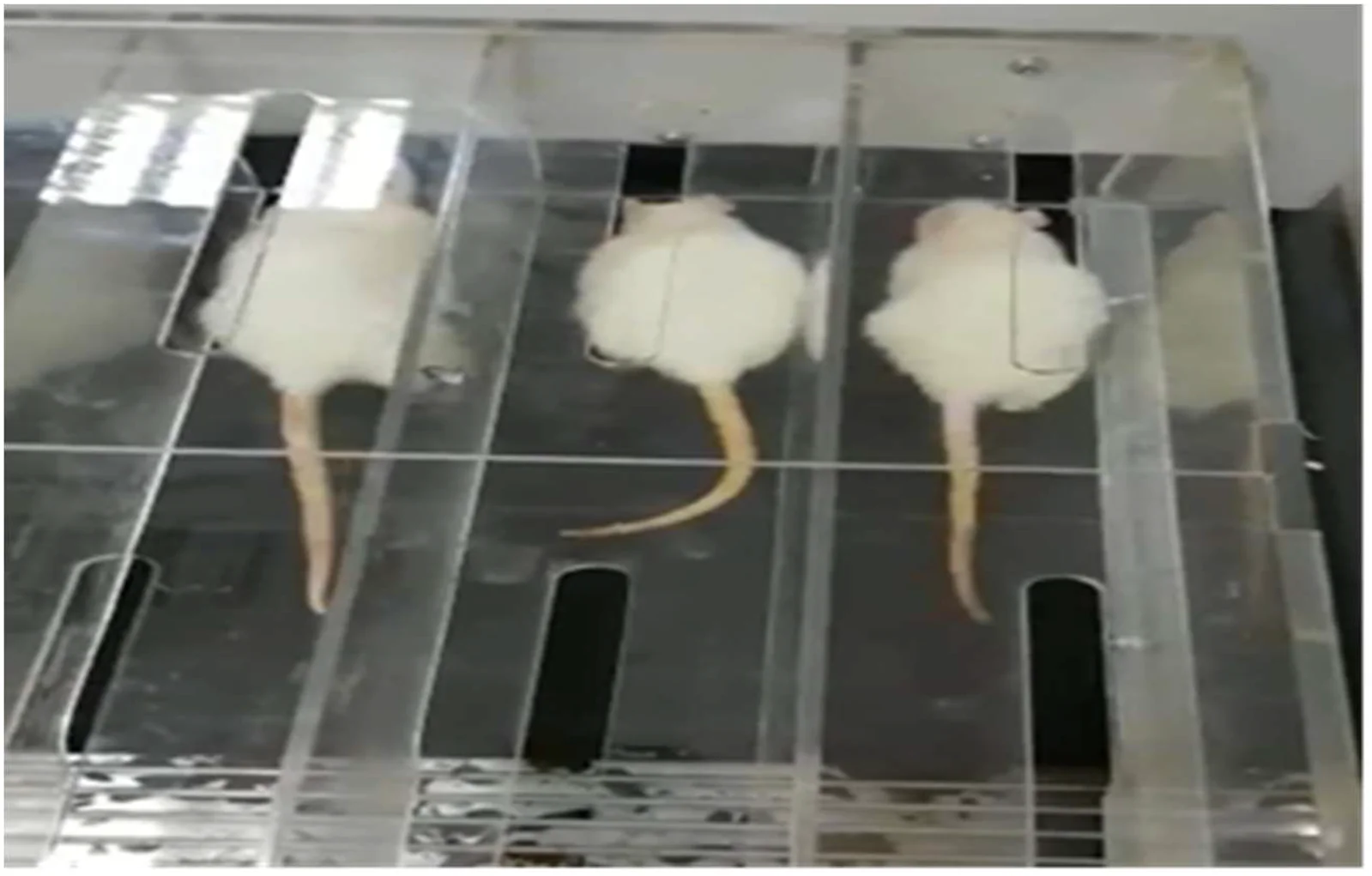
Treadmill, (ELIKMAK Medical Devices, Elazıg, Türkiye).

The exercise protocols were conducted at the GAUNDAM facility using a treadmill (ELIKMAK Medical Devices Ltd., Elazıg, Türkiye). Rats in the Control, Sham (STZ Solvent), Diabetes, and Diabetes+Supplement groups did not participate in any exercise interventions and served as non-exercising controls. For the remaining groups, the first week (5 days) was designated as an adaptation phase to acclimate the rats to treadmill running. This adaptation protocol, based on previous studies involving treadmill exercise in rats, recommended low-intensity running to start (Ni et al., 2013; Zeng et al., 2021). During this phase, the treadmill was set to 10 m/min with no incline (0°), and the rats ran for 10 minutes per day over 5 consecutive days. This protocol allowed the rats to become accustomed to the treadmill environment while minimising stress-related confounding factors (**Figure 3**).

Following the adaptation period, exercise protocols were implemented for the remaining groups. These sessions took place on weekdays between 10:00 and 12:00. The exercise protocols were structured to reflect the respective study objectives, and were adjusted for intensity and duration based on the experimental group assignments. Detailed descriptions of the exercise regimens are illustrated in **Table 1**. The intensity levels were defined based on treadmill speed in accordance with previously published rat exercise protocols: 10 m/min was classified as low intensity, 17 m/min as moderate intensity, and 22 m/min as high intensity, consistent with the protocols reported by Wang et al. (2020) and Heo et al. (2021). All exercise sessions were conducted under identical environmental conditions (22 ± 3 °C) on the same treadmill device. .

**Table 1:** Exercise protocols for experimental animals.

Week	Exercise 1 (Low Intensity)	Exercise 2 (Moderate Intensity)	Exercise 3 (High Intensity)
Week 1 Adaptation	10 m/min/10 min	10 m/min/10 min	10 m/min/10 min
Week 2	10 m/min/10 min	10 m/min/15 min	10 m/min/15 min
Week 3	10 m/min/15 min	10 m/min/15 min 17 m/min/5 min	17 m/min/15 min
Week 4	10 m/min/15 min	10 m/min/15 min 17 m/min/10 min	17 m/min/15 min
Week 5	10 m/min/15 min	10 m/min/15 min 17 m/min/15 min	17 m/min/15 min 22 m/min/10 min

### Experiment completion and blood sample collection

At the conclusion of Week 5, all experimental animals were euthanised by decapitation using a guillotine between 9:00 AM and 10:00 AM, one day after the final exercise session, in accordance with the approved ethical protocol. Blood samples were then collected from the jugular vein and transferred into biochemistry tubes. These samples were centrifuged at 4000 rpm for 10 minutes at the animal research center to separate serum, which was stored at -80 °C until analysis. Serum levels of adiponectin, betatrophin, irisin, TNF-α, IL-1, and IL-6 were measured using commercial kits for rats and analysed via Enzyme-Linked Immunosorbent Assay (ELISA) method. ELISA procedures were carried out in accordance with the manufacturer’s protocols, with measurements taken using an ELISA Reader (Biotek, H1 Synergy, USA). The concentrations of the samples were calculated using a standard curve generated from the standards included in the kit. Insulin, AST, and ALT levels were analysed at the Central Laboratory of Gaziantep University Research Hospital using an Abbott Architect C8000 analyzer. Although full blinding of exercise group allocation was not feasible given the nature of the multi-group treadmill protocol, assessor blinding was implemented for biochemical outcome measurement: all ELISA analyses and clinical chemistry assays were performed by laboratory personnel who were unaware of the animals’ group assignments. Prior to euthanasia, animals were fasted for approximately 2 hours to minimize the acute effects of food intake on biochemical parameters, particularly glucose and insulin levels. Although this fasting period is shorter than the overnight fasting typically used in metabolic studies, it was considered sufficient given the severity of hyperglycemia in STZ-induced diabetic rats, in which prolonged fasting may cause additional metabolic stress and compromise animal welfare.

The HOMA (Homeostatic Model Assessment) index was not calculated in this study, as HOMA-IR is primarily validated for assessing insulin resistance in type 2 diabetes models with residual beta-cell function. In the present STZ-induced type 1 diabetes model, pancreatic beta cells are substantially destroyed, resulting in severely reduced endogenous insulin secretion. Under these conditions, HOMA-IR values would not reliably reflect insulin resistance and their clinical interpretation would be limited.

### Statistical analysis

Data analysis was conducted using SPSS software (version 22.0, SPSS Inc., Chicago, IL, USA). Data are presented as mean ± standard deviation. Normality was assessed using the Shapiro-Wilk test, and homogeneity of variances was evaluated using the Levene test. For datasets deviating from normality, skewness and kurtosis values were examined; values within ±2 were considered acceptable for parametric analysis. Within-group differences between pre- and post-test values were assessed using paired samples t-test. Between-group differences were analysed using one-way ANOVA, and Tukey’s *post hoc* test was applied to identify pairwise differences. Effect sizes were calculated using Cohen’s d (Cohen, 1988). Statistical significance was set at p < 0.05.

## Results

The results of all statistical analyses are presented below. Data are expressed as mean ± standard deviation. Statistically significant differences between groups are indicated by superscript letters in each table (p < 0.05). .

Within-group comparisons revealed significant increases in body weight in the control and sham groups (Groups 1 and 2) and in the combined ACV and low- to moderate-intensity exercise groups (Groups 8 and 9) (p < 0.05). The diabetes group (Group 3) exhibited significant weight loss over the experimental period (p = 0.002, Cohen’s d = 2.06), consistent with the catabolic effects of STZ-induced hyperglycemia. The Diabetes + ACV + Exercise 3 group (Group 10) also showed significant weight loss (p = 0.008, Cohen’s d = 1.29), suggesting that high-intensity exercise may exacerbate weight loss in diabetic conditions even when combined with ACV. No significant within-group change in body weight was observed in the Diabetes + Exercise groups (Groups 5, 6, and 7) (p > 0.05) (**Table 2**). Between-group comparisons revealed no statistically significant differences in body weight at either baseline or end of study (p > 0.05), as shown in **Table 2a**.

**Table 2:** Statistical analyses of body weight (g): within-group comparisons (paired t-test).

Group	Time	Mean ± SD	t	*p*	Cohen’s d
1. Control	Pre-test	229 ± 20	-10.3	**<0.001**	-3.63
	Post-test	306 ± 18			
2. Sham	Pre-test	218 ± 17	-13.0	**<0.001**	-4.60
	Post-test	298 ± 19			
3. Diabetes	Pre-test	231 ± 20	5.46	**0.002**	2.06
	Post-test	213 ± 16			
4. D+Supp.	Pre-test	216 ± 21	-1.33	0.227	-0.469
	Post-test	228 ± 16			
5. D+Exerc 1	Pre-test	226 ± 21	-0.287	0.783	-0.101
	Post-test	228 ± 16			
6. D+Exerc 2	Pre-test	226 ± 21	0.676	0.520	0.239
	Post-test	221 ± 17			
7. D+Exerc 3	Pre-test	217 ± 16	1.43	0.197	0.504
	Post-test	206 ± 13			
8. D+Supp+Exerc 1	Pre-test	222 ± 24	-5.61	**<0.001**	-1.98
	Post-test	244 ± 26			
9. D+Supp+Exerc 2	Pre-test	217 ± 16	-2.90	**0.023**	-1.03
	Post-test	230 ± 16			
10. D+Supp+Exerc 3	Pre-test	221 ± 17	3.64	**0.008**	1.29
	Post-test	203 ± 17			

Bold p-values indicate statistical significance (p < 0.05).

**Table 2a:** Body weight (g) at baseline and end of study: between-group comparisons (one-way ANOVA).

Group	Mean ± SD-Pre-test	Mean ± SD-Post-test
1. Control	228.75 ± 20.31*	305.63 ± 18.41
2. Sham	218.13 ± 16.89*	297.63 ± 18.88
3. Diabetes	231.43 ± 19.52*	212.86 ± 16.04
4. D+Supp.	215.63 ± 20.60	227.50 ± 15.81
5. D+Exerc 1	226.25 ± 20.66	228.13 ± 15.57
6. D+Exerc 2	226.25 ± 20.83	220.63 ± 16.57
7. D+Exerc 3	216.88 ± 15.80	205.63 ± 12.94
8. D+Supp+Exerc 1	221.88 ± 23.59*	244.38 ± 26.11
9. D+Supp+Exerc 2	216.88 ± 15.80	230 ± 16.04
10. D+Supp+Exerc 3	221.25 ± 17.27*	202.50 ± 16.69

* = Significant difference between pre- and post-test values within the group (paired t-test). No statistically significant difference was found between groups at either time point (One-Way ANOVA, p > 0.05).

**Table 2a** presents between-group comparisons of body weight at baseline and end of study. Although several groups showed significant within-group changes (indicated by), one-way ANOVA revealed no statistically significant differences between groups at either time point (p > 0.05), indicating that initial body weights were comparable across all groups and that the observed within-group changes were not significantly different between experimental conditions.

Within-group comparisons demonstrated a significant increase in fasting blood glucose levels following STZ injection in all diabetic and intervention groups (p < 0.001), confirming successful diabetes induction (**Table 3**). Between-group comparisons revealed no statistically significant differences in post-STZ glucose levels across groups (p > 0.05), as shown in **Table 3a**, indicating comparable glycemic responses to STZ induction across all experimental conditions. Cohen’s d values indicated large effect sizes across all groups (|d| > 0.8), reflecting the magnitude of STZ-induced hyperglycemia.

**Table 3:** Fasting blood glucose levels (mg/dL) before and after STZ induction: within-group comparisons (paired t-test).

Group	Time	Mean ± SD	t	p	Cohen’s d
3. Diabetes	Pre-test	89.6 ± 9.9	-8.92	**<0.001**	-3.37
	Post-test	420.4 ± 105.4			
4. D+Supp.	Pre-test	100.0 ± 10.4	-10.1	**<0.001**	-3.57
	Post-test	356.0 ± 68.1			
5. D+Exerc 1	Pre-test	96.4 ± 8.2	-9.38	**<0.001**	-3.32
	Post-test	381.9 ± 80.5			
6. D+Exerc 2	Pre-test	93.0 ± 15.5	-10.5	**<0.001**	-3.70
	Post-test	333.1 ± 58.1			
7. D+Exerc 3	Pre-test	97.6 ± 11.0	-15.4	**<0.001**	-5.45
	Post-test	323.1 ± 33.4			
8. D+Supp+Exerc 1	Pre-test	91.9 ± 7.8	-9.62	**<0.001**	-3.40
	Post-test	375.6 ± 80.2			
9. D+Supp+Exerc 2	Pre-test	95.3 ± 10.6	-12.1	**<0.001**	-4.28
	Post-test	355.4 ± 63.2			
10. D+Supp+Exerc 3	Pre-test	99.5 ± 12.0	-13.5	**<0.001**	-4.77
	Post-test	331.6 ± 47.6			

Bold p-values indicate statistical significance (p < 0.05).

**Table 3a:** Fasting blood glucose levels (mg/dL) before and after STZ induction: between-group comparisons (one-way ANOVA).

Group	Mean ± SD-Pre-test	Mean ± SD-Post-test
3. Diabetes	89.57 ± 9.88*	420.43 ± 105.38
4. D+Supp.	100.38 ± 10.43*	356 ± 68.12
5. D+Exerc 1	96.38 ± 8.21*	381.88 ± 80.49
6. D+Exerc 2	93 ± 15.49*	333.13 ± 58.06
7. D+Exerc 3	97.63 ± 10.99*	323.13 ± 33.37
8. D+Supp+Exerc 1	91.88 ± 7.79*	375.63 ± 80.24
9. D+Supp+Exerc 2	95.25 ± 10.59*	355.38 ± 63.16
10. D+Supp+Exerc 3	99.50 ± 12*	331.63 ± 47.58

* = Significant within-group increase after STZ induction (paired t-test, p < 0.05). No statistically significant differences were found between groups at either time point (one-way ANOVA, p > 0.05). Numerical differences between intervention groups should not be interpreted as clinically meaningful in the absence of statistical significance.

As shown in **Tables 3a**, one-way ANOVA revealed no statistically significant differences between groups at either time point (p > 0.05). Numerical differences in post-STZ glucose values between intervention groups should not be interpreted as clinically meaningful in the absence of between-group statistical significance.

**Figure 4:**
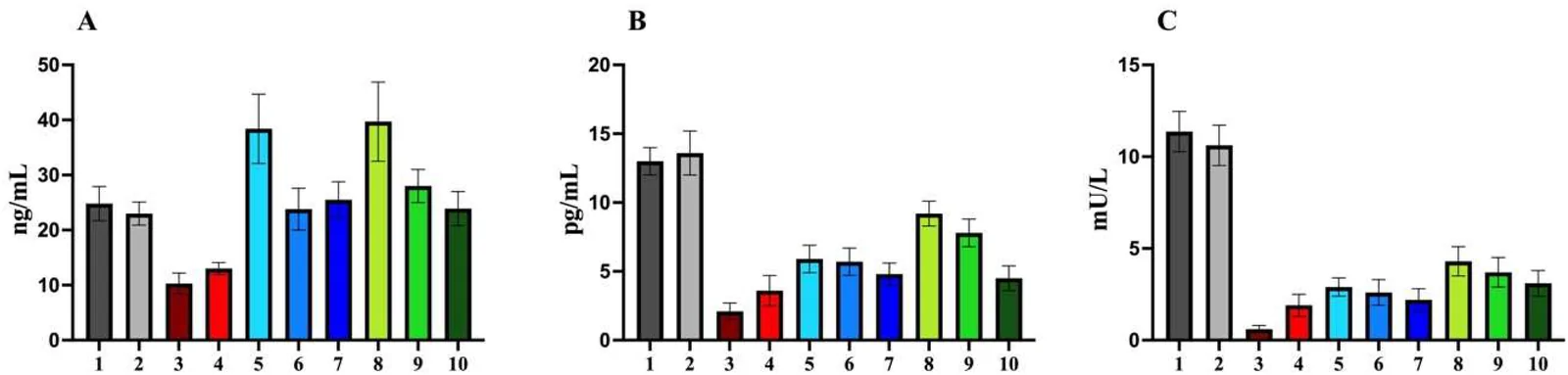
Serum metabolic hormone levels across the experimental groups. **(A)** Serum irisin, **(B)** serum adiponectin, and **(C)** serum insulin levels. Data are presented as mean ± SD. Groups: 1, Control; 2, Sham (STZ solvent); 3, Diabetes; 4, Diabetes + ACV; 5, Diabetes + low-intensity exercise; 6, Diabetes + moderate-intensity exercise; 7, Diabetes + high-intensity exercise; 8, Diabetes + ACV + low-intensity exercise; 9, Diabetes + ACV + moderate-intensity exercise; and 10, Diabetes + ACV + high-intensity exercise. The final sample size was n = 8 per group, except for the Diabetes group (n = 7), ACV, apple cider vinegar.

**Figure 5:**
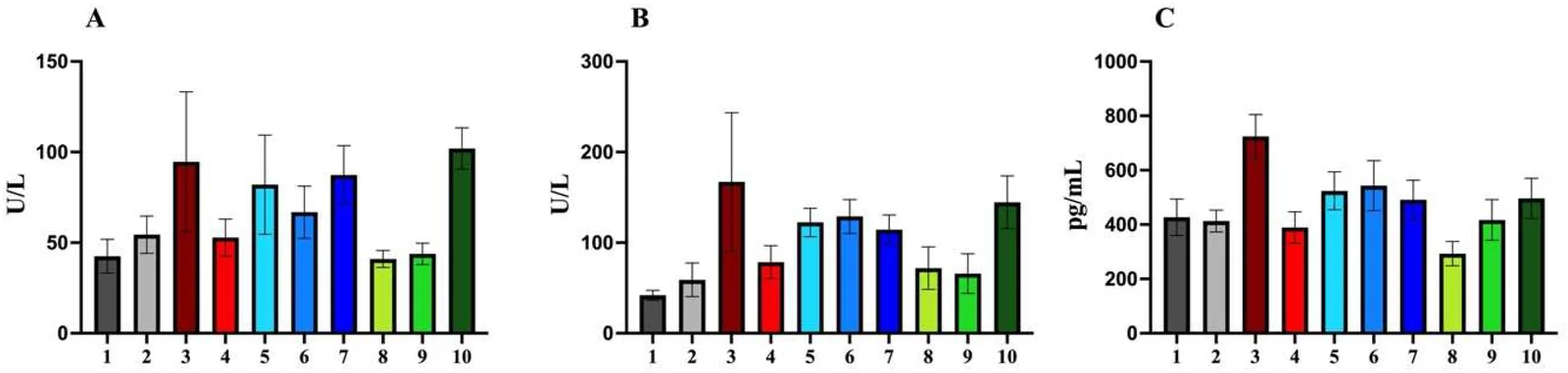
Serum hepatic and metabolic marker levels across the experimental groups. **(A)** Serum alanine aminotransferase (ALT), **(B)** serum aspartate aminotransferase (AST), and **(C)** serum betatrophin (ANGPTL8) levels. Data are presented as mean ± SD. Groups: 1, Control; 2, Sham (STZ solvent); 3, Diabetes; 4, Diabetes + ACV; 5, Diabetes + low-intensity exercise; 6, Diabetes + moderate-intensity exercise; 7, Diabetes + high-intensity exercise; 8, Diabetes + ACV + low-intensity exercise; 9, Diabetes + ACV + moderate-intensity exercise; and 10, Diabetes + ACV + high-intensity exercise. The final sample size was n = 8 per group, except for the Diabetes group (n = 7), ACV, apple cider vinegar.

**Figure 6:**
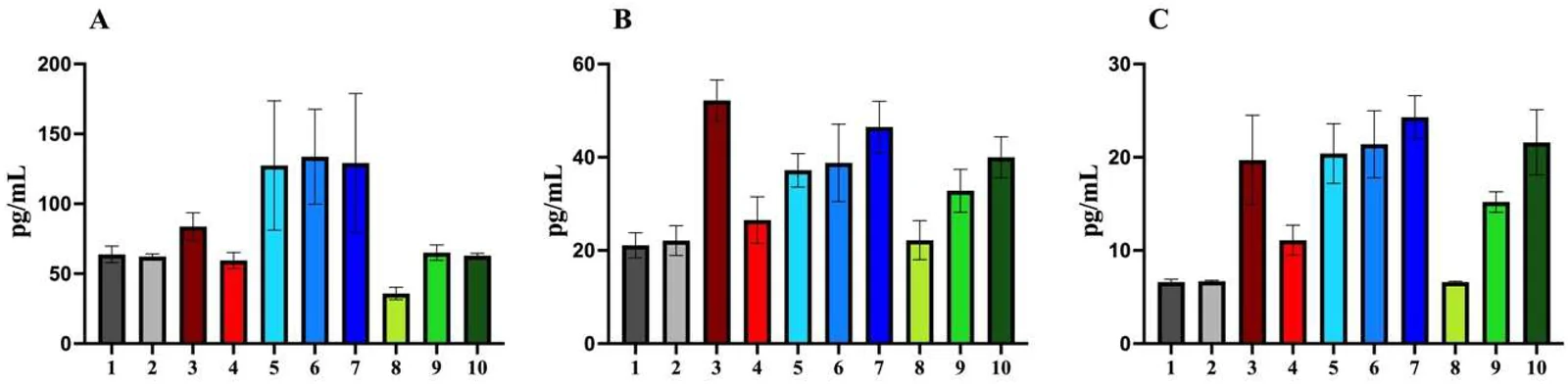
Serum cytokine levels across the experimental groups. **(A)** Serum IL-1β, **(B)** serum IL-6, and **(C)** serum TNF-α levels. Data are presented as mean ± SD. Groups: 1, Control; 2, Sham (STZ solvent); 3, Diabetes; 4, Diabetes + ACV; 5, Diabetes + low-intensity exercise; 6, Diabetes + moderate-intensity exercise; 7, Diabetes + high-intensity exercise; 8, Diabetes + ACV + low-intensity exercise; 9, Diabetes + ACV + moderate-intensity exercise; and 10, Diabetes + ACV + high-intensity exercise. The final sample size was n = 8 per group, except for the Diabetes group (n = 7), ACV, apple cider vinegar.

**Table 4** presents between-group comparisons of irisin, adiponectin, and insulin levels. Statistically significant differences are indicated by superscript letters (p < 0.05); see table legend for details (**Figures 4**–**6**).

**Table 4:** Serum irisin (ng/mL), adiponectin (µg/mL), and insulin (µIU/mL) levels across groups (mean ± SD; one-way ANOVA with Tukey *post hoc* test).

Group	Irisin	Adiponectin	Insulin
1. Control	24.8 ± 3.1	13.0 ± 1.0	11.37 ± 1.1
2. Sham	23.0 ± 2.1	13.6 ± 1.6	10.62 ± 1.1
3. Diabetes	10.3 ± 1.9 **^a,b^**	2.1 ± 0.6 ^a,b^	0.6 ± 0.2 ^a,b^
4. D+Supp.	13.0 ± 1.1 **^a,b^**	3.6 ± 1.1 ^a,b^	1.9 ± 0.6 ^a,b,c^
5. D+Exerc 1	38.4 ± 6.3 ^a,b,c,d^	5.9 ± 1.0 ^a,b,c,d^	2.9 ± 0.5 ^a,b,c^
6. D+Exerc 2	23.8 ± 3.8 ^c,d,e^	5.7 ± 1.0 ^a,b,c,d^	2.6 ± 0.7 ^a,b,c^
7. D+Exerc 3	25.5 ± 3.3 ^c,d,e^	4.8 ± 0.8 ^a,b,c^	2.2 ± 0.6 ^a,b,c,h,i^
8. D+Supp+Exerc 1	39.7 ± 7.2 ^a,b,c,d,f,g^	9.2 ± 0.9 **^a,b,c,d,e,f,g^**	4.3 ± 0.8 ^a,b,c,d,e,f,g^
9. D+Supp+Exerc 2	28.0 ± 3.0 ^c,d,e,h^	7.8 ± 1.0 ^a,b,c,d,e,f,g^	3.7 ± 0.8 ^a,b,c,d^
10. D+Supp+Exerc 3	23.9 ± 3.1 ^c,d,e,h^	4.5 ± 0.9 ^a,b,c,h,i^	3.1 ± 0.7 ^a,b,c^

^*a*^Statistically significant difference compared to the Control group. ^*b*^Statistically significant difference compared to the Sham group. ^*c*^Statistically significant difference compared to the Diabetes group. ^*d*^Statistically significant difference compared to the Diabetes + ACV group. ^*e*^Statistically significant difference compared to the Diabetes + Exercise 1 group. ^*f*^Statistically significant difference compared to the Diabetes + Exercise 2 group. ^*g*^, Statistically significant difference compared to the Diabetes + Exercise 3 group. ^*h*^, Statistically significant difference compared to the Diabetes + ACV + Exercise 1 group. ^*i*^, Statistically significant difference compared to the Diabetes + ACV + Exercise 2 group. All comparisons based on one-way ANOVA with Tukey *post hoc* test (p < 0.05). Bold p-values indicate statistical significance (p < 0.05).

## Discussion

This study investigated the combined effects of ACV supplementation and treadmill exercise at varying intensities on metabolic and inflammatory biomarkers in STZ-induced diabetic rats. STZ treatment resulted in significant weight loss in the diabetes group (p = 0.002), consistent with the catabolic effects of chronic hyperglycemia. Although ACV supplementation and exercise interventions showed numerical trends toward weight stabilization, between-group comparisons revealed no statistically significant differences in body weight across groups (p > 0.05). The combination of ACV with low- and moderate-intensity exercise (Groups 8 and 9) was associated with significant within-group weight gain, suggesting a potentially beneficial interaction that warrants further investigation in future studies.

The acetic acid and polyphenol content of ACV have been proposed to attenuate catabolic processes in diabetic models via suppression of oxidative stress and NF-κB-mediated inflammation (Budak et al., 2011; Bouderbala et al., 2016; Jin et al., 2023). However, as between-group differences in body weight were not statistically significant in the present study, mechanistic conclusions regarding the weight-modulating effects of these interventions cannot be drawn from the available data.

Following STZ induction, fasting blood glucose levels were significantly elevated in all diabetic groups (p < 0.001), confirming successful diabetes induction. Between-group comparisons revealed no statistically significant differences in post-STZ glucose levels (p > 0.05), indicating that neither ACV nor exercise, alone or in combination, produced a statistically significant reduction in fasting blood glucose under the present experimental conditions. This finding may reflect the severity of STZ-induced beta-cell destruction in the type 1 model, which substantially eliminates endogenous insulin secretion and may therefore be less responsive to short-term dietary or exercise interventions than type 2 models with residual beta-cell function (Damasceno et al., 2014; Arjmandfard et al., 2025).

STZ induction resulted in a dramatic reduction in fasting insulin levels in the diabetes group compared to controls (p < 0.05). Both ACV supplementation and exercise interventions significantly improved insulin levels relative to the diabetes group, with the greatest improvement in Group 8 (ACV + low-intensity exercise, p < 0.05). The present design does not permit formal interaction testing; therefore, the term synergism is avoided. Regarding irisin, low- and moderate-intensity exercise, particularly when combined with ACV, significantly increased irisin levels compared to the diabetes group (p < 0.05). Mechanistically, skeletal muscle contraction upregulates PGC-1α, which drives proteolytic cleavage of FNDC5 to release irisin; this pathway is suppressed in STZ-induced diabetic models through oxidative stress-mediated inhibition of FNDC5 expression (Behera et al., 2022; Wang et al., 2023). The polyphenolic compounds in ACV may partially restore this pathway by reducing reactive oxygen species and attenuating oxidative suppression of PGC-1α signalling (Budak et al., 2011; Khalafi et al., 2016). High-intensity exercise groups showed irisin levels significantly above the diabetes group but significantly below the low-intensity combined group, a pattern consistent with ROS-mediated attenuation of FNDC5 expression under high oxidative load (Radak et al., 2008; Leal et al., 2018).

Adiponectin was significantly reduced in the diabetes group relative to controls (p < 0.05) and showed the greatest statistically significant recovery in Group 8 (ACV + low-intensity exercise, p < 0.05 vs. diabetes). Adiponectin exerts its insulin-sensitizing effects primarily through AdipoR1/R2-mediated activation of the AMPK–PPARα pathway, which is suppressed in insulin-deficient states (Yamauchi and Kadowaki, 2013; Abdalla, 2024). Low- and moderate-intensity exercise stimulates AMPK and upregulates PPAR-γ expression in adipose tissue, supporting adiponectin synthesis; ACV polyphenols may reinforce this effect by suppressing NF-κB-driven inflammation that inhibits adiponectin secretion (Budak et al., 2011; Kandylis et al., 2021). High-intensity exercise, despite ACV co-administration, was associated with comparatively lower adiponectin levels among exercising groups, consistent with ROS-mediated suppression of adiponectin at high oxidative loads (Radak et al., 2008).

Betatrophin (ANGPTL8), expressed predominantly in the liver and adipose tissue, is implicated in lipid metabolism regulation and has been proposed as a potential target in metabolic disorders including diabetes and metabolic syndrome (Abu-Farha et al., 2016; Sadeghipour et al., 2023; Hu et al., 2019). In the present study, betatrophin levels were significantly elevated in diabetic rats compared to controls (p < 0.05), consistent with reports of increased betatrophin in insulin-resistant states (Guo et al., 2022). ACV supplementation alone and in combination with exercise significantly reduced betatrophin levels toward control values, with the greatest reduction observed in the ACV combined with low-intensity exercise group (Group 8, 293 ± 45 pg/mL) (**Table 5**). These findings suggest that ACV and low-to-moderate intensity exercise may beneficially modulate betatrophin-associated lipid dysregulation in diabetic conditions. Betatrophin regulates lipid metabolism partly through inhibition of lipoprotein lipase activity, and elevated levels in diabetic states may reflect a compensatory response to insulin resistance and dyslipidemia (Amri et al., 2019; Mahmoudi et al., 2019). In the present study, the significant reduction in betatrophin levels observed with ACV supplementation may be attributed to the lipid-regulating effects of its polyphenols and organic acids, which reduce hepatic lipid accumulation and enhance fatty acid oxidation (Amri et al., 2019). Similarly, low- and moderate-intensity exercise may reduce betatrophin through AMPK-mediated improvements in lipid metabolism and insulin sensitivity (Sadeghipour et al., 2023; Wang et al., 2020; Lin et al., 2024). High-intensity exercise combined with ACV (Group 10) showed less favorable betatrophin reduction compared to low-intensity combinations, consistent with the hypothesis that oxidative stress induced by intense exercise may partially counteract the beneficial metabolic effects of ACV (Radak et al., 2008).

**Table 5:** Serum ALT (U/L), AST (U/L), and betatrophin (pg/mL) levels across groups (mean ± SD; one-way ANOVA with Tukey *post hoc* test).

Group	ALT	AST	Betatrophin
1. Control	42.5 ± 9.3	41.9 ± 5.6	427 ± 67
2. Sham	54.4 ± 10.3	58.9 ± 18.5	413 ± 40
3. Diabetes	94.6 ± 38.6 ^a,b,d,h,i^	167.1 ± 76.4 ^a,b^	724 ± 81 ^a,b^
4. D+Supp.	52.8 ± 10.2	78.4 ± 18.2 ^c^	389 ± 58 ^c^
5. D+Exerc 1	82 ± 27.4 ^a,d^	122.3 ± 15.7 ^a,b,c^	524 ± 70 ^c,d^
6. D+Exerc 2	66.8 ± 14.4	128.9 ± 18.7 ^a,b,d^	543 ± 92 ^a,b,c,d^
7. D+Exerc 3	87.3 ± 16.3 ^a,b,d^	114.3 ± 16.4 ^a,b,c^	491 ± 72 ^c^
8. D+Supp+Exerc 1	41.0 ± 4.7 ^e,g^	71.8 ± 23.5 ^c,e,f^	293 ± 45 ^a,b,c,e,f,g^
9. D+Supp+Exerc 2	43.8 ± 5.9 ^e,g^	65.9 ± 21.9 ^c,e,f,g^	417 ± 75 ^c,f,h^
10. D+Supp+Exerc 3	102.0 ± 11.4 ^a,b,d,f,h,i^	144.6 ± 29.0^a,b,d,h,i^	496 ± 74 ^c,h^

^*a*^Statistically significant difference compared to the Control group. ^*b*^Statistically significant difference compared to the Sham group. ^*c*^Statistically significant difference compared to the Diabetes group. ^*d*^Statistically significant difference compared to the Diabetes + ACV group. ^*e*^Statistically significant difference compared to the Diabetes + Exercise 1 group. ^*f*^Statistically significant difference compared to the Diabetes + Exercise 2 group. ^*g*^Statistically significant difference compared to the Diabetes + Exercise 3 group. ^*h*^Statistically significant difference compared to the Diabetes + ACV + Exercise 1 group. ^*i*^Statistically significant difference compared to the Diabetes + ACV + Exercise 2 group. All comparisons based on one-way ANOVA with Tukey *post hoc* test (p < 0.05).

The present findings suggest that the elevated betatrophin observed in STZ-induced diabetes reflects a compensatory response to severe metabolic dysregulation, and that ACV combined with low-intensity exercise most effectively normalizes this response. Future studies should investigate the mechanistic link between betatrophin, AMPK activation, and ACV bioactive compounds in greater detail, as well as the long-term effects of different exercise intensities on betatrophin regulation in diabetic models (Guo et al., 2022).

AST and ALT are established markers of hepatic stress, and their significant elevation in the diabetes group (Group 3) is consistent with STZ-induced liver damage reported in the literature (Amirhosravi et al., 2023; Halima et al., 2018; Ousaaid et al., 2020). ACV supplementation alone significantly reduced both enzymes compared to the diabetes group, supporting its hepatoprotective potential through free radical neutralization and reduction of oxidative damage in liver tissue (Ding et al., 2025). The combination of ACV with low-intensity exercise (Group 8) produced the greatest reductions in AST and ALT, approaching control values. However, high-intensity exercise combined with ACV (Group 10) showed paradoxically elevated AST and ALT levels, suggesting that excessive exercise load may exacerbate hepatic stress even in the presence of ACV supplementation. This intensity-dependent pattern underscores the importance of exercise prescription in diabetic individuals with compromised liver function. Exercise may further support liver health by reducing hepatic lipid accumulation, while ACV controls oxidative stress and inflammation through its bioactive compounds (Kumar et al., 2019). These complementary mechanisms may explain the superior hepatoprotective effect observed in the ACV combined with low-intensity exercise group. In contrast, high-intensity exercise groups showed no improvement in AST and ALT levels, consistent with reports that intense exercise increases free radical production and triggers a hepatic stress response (Radak et al., 2008). These findings suggest that exercise intensity is a critical determinant of hepatic outcomes in diabetic conditions, and that moderate-to-low intensity protocols are preferable for supporting liver health. Future studies should examine the long-term hepatic effects of combined ACV and exercise interventions across different diabetic models.

Pro-inflammatory cytokines including IL-1β, IL-6, and TNF-α play a central role in diabetes-associated inflammation and are closely linked to insulin resistance and metabolic disorders (Chen et al., 2017; Uno et al., 2007; Alfadul et al., 2022; Xia et al., 2023). In the present study, the combination of ACV with low-intensity exercise (Group 8) produced the most pronounced reductions in all three cytokines, with TNF-α reaching control-level values (6.6 ± 0.1 pg/mL) (**Table 6**). These findings are consistent with reports highlighting the anti-inflammatory effects of dietary polyphenols through suppression of the NF-κB pathway and reduction of oxidative stress (Zhu et al., 2020; Alfadul et al., 2022). The phenolic compounds and organic acids in ACV may thus complement exercise-induced anti-inflammatory adaptations, contributing to improved cytokine regulation in diabetic conditions.

**Table 6:** Serum IL-1β (pg/mL), IL-6 (pg/mL), and TNF-α (pg/mL) levels across groups (mean ± SD; one-way ANOVA with Tukey *post hoc* test).

Group	IL-1β	IL-6	TNF-α
1. Control	63.8 ± 5.9	21.1 ± 2.7	6.6 ± 0.3
2. Sham	62.3 ± 1.9	22.1 ± 3.2	6.7 ± 0.1
3. Diabetes	83.7 ± 9.9	52.2 ± 4.4 ^a,b^	19.7 ± 4.8 ^a,b^
4. D+Supp.	59.5 ± 5.7	26.5 ± 5.0 ^c^	11.1 ± 1.6 ^a,b,c^
5. D+Exerc 1	127.4 ± 46.2 ^a,b,c,d^	37.2 ± 3.6 ^a,b,c,d^	20.4 ± 3.2 ^a,b,d^
6. D+Exerc 2	133.7 ± 33.9 ^a,b,c,d^	38.8 ± 8.3 ^a,b,c,d^	21.4 ± 3.6 ^a,b,d^
7. D+Exerc 3	129.2 ± 49.7 ^a,b,c,d^	46.5 ± 5.5 ^a,b,d,e^	24.3 ± 2.3 ^a,b,c,d^
8. D+Supp+Exerc 1	35.9 ± 4.4 ^c,e,f,g^	22.2 ± 4.2 ^c,e,f,g^	6.6 ± 0.1^c,d,e,f,g^
9. D+Supp+Exerc 2	65.1 ± 5.5 ^e,f,g^	32.8 ± 4.6 ^a,b,c,g,h^	15.2 ± 1.1 ^a,b,c,e,f,g,h^
10. D+Supp+Exerc 3	62.9 ± 1.6 ^e,f,g^	40.0 ± 4.4 ^a,b,c,d,h^	21.6 ± 3.5 ^a,b,d,h,i^

^*a*^Statistically significant difference compared to the Control group. ^*b*^Statistically significant difference compared to the Sham group. ^*c*^Statistically significant difference compared to the Diabetes group. ^*d*^Statistically significant difference compared to the Diabetes + ACV group. ^*e*^Statistically significant difference compared to the Diabetes + Exercise 1 group. ^*f*^Statistically significant difference compared to the Diabetes + Exercise 2 group. ^*g*^Statistically significant difference compared to the Diabetes + Exercise 3 group. ^*h*^Statistically significant difference compared to the Diabetes + ACV + Exercise 1 group. ^*i*^Statistically significant difference compared to the Diabetes + ACV + Exercise 2 group. All comparisons based on one-way ANOVA with Tukey *post hoc* test (p < 0.05).

The dual role of IL-6 as both pro- and anti-inflammatory mediator is well established; during moderate-intensity exercise, muscle-derived IL-6 acts in an anti-inflammatory capacity by suppressing TNF-α and promoting IL-10 release (Isanejad et al., 2015; Moon et al., 2012; Koelman et al., 2019; Petersen and Pedersen, 2005). This mechanism may partly explain the cytokine reductions observed in low- and moderate-intensity combined groups in the present study. Notably, IL-1β levels were paradoxically elevated in exercise-only groups (Groups 5, 6, and 7) compared to both control and diabetes groups, suggesting that treadmill exercise in STZ-induced diabetic rats may trigger an acute inflammatory response independent of ACV supplementation. This finding is consistent with reports that intense or prolonged exercise increases pro-inflammatory cytokine production through oxidative stress mechanisms (Radak et al., 2008), and warrants further investigation in future studies. In contrast, when ACV was combined with exercise, IL-1β levels were substantially reduced, suggesting that the antioxidant properties of ACV may attenuate exercise-induced inflammatory responses in diabetic conditions.

Several limitations of the present study should be acknowledged. First, the four-week intervention period may not be sufficient to capture the full spectrum of metabolic adaptations to ACV and exercise, and longer protocols are warranted in future studies. Second, the STZ-induced type 1 diabetes model substantially destroys pancreatic beta cells and therefore does not fully replicate the metabolic complexity of human diabetes; findings should be interpreted with caution regarding clinical translation. Third, the study relied exclusively on circulating serum biomarkers and did not include tissue-level analyses. Biopsies from key metabolically active tissues such as skeletal muscle, liver, and adipose tissue would have provided more direct mechanistic insight into the observed changes. Fourth, although the bioactive composition of ACV was verified by GC-MS prior to the experiment, longitudinal stability of the preparation was not assessed; batch-to-batch and storage-related variability in bioactive content cannot be excluded. Fifth, the absence of a positive treatment control (e.g., metformin or insulin) limits the ability to benchmark the magnitude of observed effects against standard-of-care interventions. Future studies should address these limitations through longer intervention periods, multi-tissue molecular analyses, inclusion of positive controls, and validation in models that more closely approximate human diabetes.

## Conclusion

From a translational perspective, the present findings should be interpreted as preliminary, hypothesis-generating evidence rather than a basis for clinical recommendations. ACV is a widely available, low-cost functional food with an established safety profile at the doses used; however, the STZ-induced type 1 rat model used here does not replicate the hormonal, metabolic, or immunological complexity of human diabetes. The observed improvements in circulating biomarkers are encouraging and provide mechanistic rationale for further investigation, but direct extrapolation to human clinical outcomes is not warranted at this stage. Future clinical studies should examine whether ACV supplementation combined with structured low-to-moderate intensity aerobic exercise produces comparable improvements in metabolic and inflammatory biomarkers in individuals with type 1 or type 2 diabetes, using validated outcome measures and appropriate controls. If confirmed in human trials, ACV could represent a low-cost, accessible adjunct dietary strategy, comparable to other evidence-based nutraceuticals such as omega-3 fatty acids or berberine, warranting integration into broader lifestyle-based diabetes management frameworks.

In this study, STZ-induced diabetes significantly impaired adiponectin, irisin, insulin, betatrophin, AST, ALT, IL-6, and TNF-α levels compared to controls. ACV supplementation alone produced statistically significant improvements in several of these biomarkers. The combination of ACV with low-intensity treadmill exercise was associated with the most favorable biomarker profile, including the greatest statistically significant reductions in inflammatory cytokines and hepatic enzymes and the highest adiponectin and insulin levels among diabetic groups. High-intensity exercise, particularly in combination with ACV, was associated with less favorable or paradoxical changes in hepatic and inflammatory markers relative to low-intensity combinations. These findings indicate that, in this STZ-induced rat model, ACV combined with low-intensity exercise modulates metabolic and inflammatory biomarkers more effectively than higher-intensity protocols. Whether these results translate to clinical settings requires confirmation in human studies, and no conclusions regarding synergistic effects or therapeutic recommendations can be drawn in the absence of formal interaction testing and clinical validation.

## Data Availability

The original contributions presented in the study are included in the article/supplementary material. Further inquiries can be directed to the corresponding author.

## References

[B1] Abdalla M. M. I. (2024). Therapeutic potential of adiponectin in prediabetes: Strategies, challenges, and future directions. Ther. Adv. Endocrinol. Metab. 15, 20420188231222371. doi: 10.1177/20420188231222371 38250316 PMC10798122

[B2] Abu-Farha M. Abubaker J. Al-Khairi I. Cherian P. Noronha F. Kavalakatt S. . (2016). Circulating angiopoietin-like protein 8 (betatrophin) association with hsCRP and metabolic syndrome. Cardiovasc. Diabetol. 15, 25. doi: 10.1186/s12933-016-0346-0 26850725 PMC4743238

[B3] Alfadul H. Sabico S. Al-Daghri N. M. (2022). The role of interleukin-1β in type 2 diabetes mellitus: A systematic review and meta-analysis. Front. Endocrinol. 13, 901616. doi: 10.3389/fendo.2022.901616 35966098 PMC9363617

[B4] Amirkhosravi L. Kordestani Z. Nikooei R. Safi Z. Yeganeh-Hajahmadi M. Mirtajaddini-Goki M. (2023). Exercise-related alterations in MCT1 and GLUT4 expressions in the liver and pancreas of rats with STZ-induced diabetes. J. Diabetes Metab. Disord. 22, 1355–1363. doi: 10.1007/s40200-023-01255-9 37975118 PMC10638214

[B5] Amri J. Parastesh M. Sadeghi M. Latifi S. A. Alaee M. (2019). High-intensity interval training improved fasting blood glucose and lipid profiles in type 2 diabetic rats more than endurance training: Possible involvement of irisin and betatrophin. Phys. Int. 106, 213–224. doi: 10.1556/2060.106.2019.24 31578075

[B6] Arjmandfard D. Behzadi M. Sohrabi Z. Mohammadi Sartang M. (2025). Effects of apple cider vinegar on glycemic control and insulin sensitivity in patients with type 2 diabetes: A GRADE-assessed systematic review and dose-response meta-analysis of controlled clinical trials. Front. Nutr. 12, 1528383. doi: 10.3389/fnut.2025.1528383 39949546 PMC11821484

[B7] Behera J. Ison J. Voor M. J. Tyagi N. (2022). Exercise-linked skeletal irisin ameliorates diabetes-associated osteoporosis by inhibiting the oxidative damage-dependent miR-150-FNDC5/pyroptosis axis. Diabetes 71, 2777–2792. doi: 10.2337/db21-0573 35802043 PMC9750954

[B8] Bouderbala H. Kaddouri H. Kheroua O. Saidi D. (2016). Anti-obesogenic effect of apple cider vinegar in rats subjected to a high fat diet. Annales. Cardiologie. d'Angéiologie. 65, 208–213. doi: 10.1016/j.ancard.2016.04.004 27209492

[B9] Bouderbala H. Kaddouri H. Maharrar M. Kheroua O. Saidi D. (2015). P2-11: Anti obesogenic effects of apple cider vinegar in rats subjected to a high fat diet. Annales. Cardiologie. d'Angéiologie. 64, S27. doi: 10.1016/S0003-3928(16)30058-0 27209492

[B10] Budak N. H. Kumbul Doguc D. Savas C. M. Seydim A. C. Kok Tas T. Ciris M. I. . (2011). Effects of apple cider vinegars produced with different techniques on blood lipids in high-cholesterol-fed rats. J. Agric. Food. Chem. 59, 6638–6644. doi: 10.1021/jf104912h 21561165

[B11] Chen Y. L. Qiao Y. C. Pan Y. H. Xu Y. Huang Y. C. Wang Y. H. . (2017). Correlation between serum interleukin-6 level and type 1 diabetes mellitus: A systematic review and meta-analysis. Cytokine 94, 14–20. doi: 10.1016/j.cyto.2017.01.002 28283222

[B12] Cohen J. (1988). Statistical Power Analysis for the Behavioral Sciences. 2nd ed (New York: Lawrence Erlbaum Associates).

[B13] Damasceno D. C. Netto A. O. Lessi I. L. Gallego F. Q. Corvino S. B. Dallaqua B. . (2014). Streptozotocin-induced diabetes models: Pathophysiological mechanisms and fetal outcomes. BioMed. Res. Int. 2014, 819065. doi: 10.1155/2014/819065 24977161 PMC4058231

[B14] Ding Q. Xue D. Ren Y. Xue Y. Shi J. Xu Z. . (2025). Apple cider vinegar powder mitigates liver injury in high-fat-diet mice via gut microbiota and metabolome remodeling. Nutrients. 17(13), 2157. doi: 10.3390/nu17132157 40647261 PMC12251902

[B15] Guan Y. Zuo F. Zhao J. Nian X. Shi L. Xu Y. . (2023). Relationships of adiponectin to regional adiposity, insulin sensitivity, serum lipids, and inflammatory markers in sedentary and endurance-trained Japanese young women. Front. Endocrinol. 14, 1097034. doi: 10.3389/fendo.2023.1097034 36761190 PMC9902352

[B16] Guo Q. Cao S. Wang X. (2022). Betatrophin and insulin resistance. Metabolites 12, 925. doi: 10.3390/metabo12100925 36295827 PMC9610572

[B17] Guo W. Peng J. Su J. Xia J. Deng W. Li P. . (2024). The role and underlying mechanisms of irisin in exercise-mediated cardiovascular protection. PeerJ 12, e18413. doi: 10.7717/peerj.18413 39494293 PMC11531754

[B18] Hadi A. Pourmasoumi M. Najafgholizadeh A. Clark C. C. T. Esmaillzadeh A. (2021). The effect of apple cider vinegar on lipid profiles and glycemic parameters: a systematic review and meta-analysis of randomized clinical trials. BMC Complement. Med. Ther. 21, 179. doi: 10.1186/s12906-021-03351-w 34187442 PMC8243436

[B19] Halima B. H. Sonia G. Sarra K. Houda B. J. Fethi B. S. Abdallah A. (2018). Apple cider vinegar attenuates oxidative stress and reduces the risk of obesity in high-fat-fed male Wistar rats. J. Med. Food 21, 70–80. doi: 10.1089/jmf.2017.0039 29091513

[B20] Heo J.-W. No M.-H. Cho J. Choi Y. Cho E.-J. Park D.-H. . (2021). Moderate aerobic exercise training ameliorates impairment of mitochondrial function and dynamics in skeletal muscle of high-fat diet-induced obese mice. FASEB J. 35, e21340. doi: 10.1096/fj.202002394R 33455027

[B21] Hu H. Yuan G. Wang X. Sun J. Gao Z. Zhou T. . (2019). Effects of a diet with or without physical activity on angiopoietin-like protein 8 concentrations in overweight/obese patients with newly diagnosed type 2 diabetes: A randomized controlled trial. Endocrine. J. 66, 89–105. doi: 10.1507/endocrj.EJ18-0191 30429410

[B22] Huang D.-D. Shi G. Jiang Y. Yao C. Zhu C. (2020). A review on the potential of resveratrol in prevention and therapy of diabetes and diabetic complications. Biomed. Pharmacotherapy. 125, 109767. doi: 10.1016/j.biopha.2019.109767 32058210

[B23] Hyun T. K. Jang K.-I. (2016). Apple as a source of dietary phytonutrients: An update on the potential health benefits of apple. EXCLI. J. 15, 565–569. doi: 10.17179/excli2016-483 28096786 PMC5225682

[B24] International Diabetes Federation (2019). IDF Diabetes Atlas, 9th Ed (Brussels: International Diabetes Federation). Available online at: https://www.diabetesatlas.org (Accessed June 19, 2026).

[B25] Isanejad A. Saraf Z. H. Mahdavi M. Gharakhanlou R. Shamsi M. M. Paulsen G. (2015). The effect of endurance training and downhill running on the expression of IL-1β, IL-6, and TNF-α and HSP72 in rat skeletal muscle. Cytokine 73, 302–308. doi: 10.1016/j.cyto.2015.03.013 25863030

[B26] Jin Q. Liu T. Qiao Y. Liu D. Yang L. Mao H. . (2023). Oxidative stress and inflammation in diabetic nephropathy: Role of polyphenols. Front. Immunol. 14, 1185317. doi: 10.3389/fimmu.2023.1185317 37545494 PMC10401049

[B27] Kandylis P. Bekatorou A. Dimitrellou D. Plioni I. Giannopoulou K. (2021). Health promoting properties of cereal vinegars. Foods 10, 344. doi: 10.3390/foods10020344 33562762 PMC7914830

[B28] Khalafi M. Shabkhiz F. Azali Alamdari K. Bakhtiyari A. (2016). Irisin response to two types of exercise training in type 2 diabetic male rats. J. Arak. Univ. Med. Sci. 19, 37–45.

[B29] Koelman L. Pivovarova-Ramich O. Pfeiffer A. F. H. Grune T. Aleksandrova K. (2019). Cytokines for evaluation of chronic inflammatory status in ageing research: Reliability and phenotypic characterisation. Immun. Ageing 16, 11. doi: 10.1186/s12979-019-0151-1 31139232 PMC6530020

[B30] Kumar A. S. Maiya A. G. Shastry B. A. Vaishali K. Ravishankar N. Hazari A. . (2019). Exercise and insulin resistance in type 2 diabetes mellitus: A systematic review and meta-analysis. Ann. Phys. Rehabil. Med. 62, 98–103. doi: 10.1016/j.rehab.2018.11.001 30553010

[B31] Leal L. G. Lopes M. A. Batista M. L. Jr. Jr. (2018). Physical exercise-induced myokines and muscle-adipose tissue crosstalk: A review of current knowledge and the implications for health and metabolic diseases. Front. Physiol. 9, 1307. doi: 10.3389/fphys.2018.01307 30319436 PMC6166321

[B32] Lin J. Zhang X. Sun Y. Xu H. Li N. Wang Y. . (2024). Exercise ameliorates muscular excessive mitochondrial fission, insulin resistance and inflammation in diabetic rats via irisin/AMPK activation. Sci. Rep. 14, 10658. doi: 10.1038/s41598-024-61415-6 38724553 PMC11082241

[B33] Mahmoudi Y. Gholami M. Nikbakht H. Ebrahimi K. Bakhtiyari S. (2019). The effects of continuous and high intensity interval trainings on plasma betatrophin level in diabetic rats treated with metformin. Iranian. J. Diabetes Obes. 11, 188–192. doi: 10.18502/ijdo.v11i3.2609

[B34] Małkowska P. (2024). Positive effects of physical activity on insulin signaling. Curr. Issues Mol. Biol. 46, 5167–5183. doi: 10.3390/cimb46060327 38920999 PMC11202552

[B35] Moon M. K. Cho B. J. Lee Y. J. Choi S. H. Lim S. Park K. S. . (2012). The effects of chronic exercise on the inflammatory cytokines interleukin-6 and tumor necrosis factor-α are different with age. Appl. Physiol. Nutr. Metab. 37, 631–636. doi: 10.1139/h2012-039 22554223

[B36] Morgan J. Mosawy S. (2016). The potential of apple cider vinegar in the management of type 2 diabetes. Int. J. Diabetes Res. 5, 129–134. doi: 10.5923/j.diabetes.20160506.02

[B37] Muramatsu K. Niwa M. Tamaki T. Ikutomo M. Masu Y. Hasegawa T. . (2017). Effect of streptozotocin-induced diabetes on motoneurons and muscle spindles in rats. Neurosci. Res. 115, 21–28. doi: 10.1016/j.neures.2016.10.004 27826051

[B38] Ni G.-X. Liu S.-Y. Lei L. Li Z. Zhou Y.-Z. Zhan L.-Q. (2013). Intensity-dependent effect of treadmill running on knee articular cartilage in a rat model. BioMed. Res. Int. 2013, 172392. doi: 10.1155/2013/172392 24693534 PMC3892754

[B39] Ousaaid D. Laaroussi H. Bakour M. ElGhouizi A. Aboulghazi A. Lyoussi B. . (2020). Beneficial effects of apple vinegar on hyperglycemia and hyperlipidemia in hypercaloric-fed rats. J. Diabetes Res. 2020, 9284987. doi: 10.1155/2020/9284987 32766316 PMC7374219

[B40] Petersen A. M. W. Pedersen B. K. (2005). The anti-inflammatory effect of exercise. J. Appl. Physiol. 98, 1154–1162. doi: 10.1152/japplphysiol.00164.2004 15772055

[B41] Radak Z. Chung H. Y. Goto S. (2008). Systemic adaptation to oxidative challenge induced by regular exercise. Free Radical Biol. Med. 44, 153–159. doi: 10.1016/j.freeradbiomed.2007.01.029 18191751

[B42] Sadeghipour H. R. Yeganeh G. Zar A. Salesi M. Akbarzadeh S. Bernardi M. (2023). The effect of 4-week endurance training on serum levels of irisin and betatrophin in streptozotocin-induced diabetic rats. Arch. Physiol. Biochem. 129, 575–581. doi: 10.1080/13813455.2020.1849310 33270481

[B43] Uno S. Imagawa A. Okita K. Sayama K. Moriwaki M. Iwahashi H. . (2007). Macrophages and dendritic cells infiltrating islets with or without beta cells produce tumour necrosis factor-alpha in patients with recent-onset type 1 diabetes. Diabetologia 50, 596–609. doi: 10.1007/s00125-006-0569-9 17221211

[B44] Voitenko N. V. Kruglikov I. A. Kostyuk E. P. Kostyuk P. G. (2000). Effect of streptozotocin-induced diabetes on the activity of calcium channels in rat dorsal horn neurons. Neuroscience 95, 519–524. doi: 10.1016/S0306-4522(99)00453-4 10658632

[B45] Wang R. Tian H. Guo D. Tian Q. Yao T. Kong X. (2020). Impacts of exercise intervention on various diseases in rats. J. Sport Health Sci. 9, 211–227. doi: 10.1016/j.jshs.2019.09.008 32444146 PMC7242221

[B46] Wang Y. Wang M. Wang Y. (2023). Irisin: A potentially fresh insight into the molecular mechanisms underlying vascular aging. Aging Dis. 15, 2491–2506. doi: 10.14336/AD.2023.1112 38029393 PMC11567262

[B47] Xia T. Kang C. Qiang X. Zhang X. Li S. Liang K. . (2023). Beneficial effect of vinegar consumption associated with regulating gut microbiome and metabolome. Curr. Res. Food Sci. 8, 100566. doi: 10.1016/j.crfs.2023.100566 38235496 PMC10792460

[B48] Xie J. Song W. Liang X. Zhang Q. Shi Y. Liu W. . (2020). Protective effect of quercetin on streptozotocin-induced diabetic peripheral neuropathy rats through modulating gut microbiota and reactive oxygen species level. Biomed. Pharmacotherapy. 127, 110147. doi: 10.1016/j.biopha.2020.110147 32559841

[B49] Yamauchi T. Kadowaki T. (2013). Adiponectin receptor as a key player in healthy longevity and obesity-related diseases. Cell Metab. 17, 185–196. doi: 10.1016/j.cmet.2013.01.001 23352188

[B50] Zeng Q. Isobe K. Fu L. Ohkoshi N. Ohmori H. Takekoshi K. . (2007). Effects of exercise on adiponectin and adiponectin receptor levels in rats. Life Sci. 80, 454–459. doi: 10.1016/j.lfs.2006.09.031 17070556

[B51] Zeng N. Liao T. Chen X.-Y. Yan Z.-P. Li J.-T. Ni G.-X. (2021). Treadmill running induces remodeling of the infrapatellar fat pad in an intensity-dependent manner. J. Orthopaedic. Surg. Res. 16, 354. doi: 10.1186/s13018-021-02501-7 34074301 PMC8167986

[B52] Zheng Y. Ley S. H. Hu F. B. (2018). Global aetiology and epidemiology of type 2 diabetes mellitus and its complications. Nature Reviews Endocrinology 14(2), 88–98. doi: 10.1038/nrendo.2017.151 29219149

[B53] Zhu S. Guan L. Tan X. Li G. Sun C. Gao M. . (2020). Hepatoprotective effect and molecular mechanisms of Hengshun aromatic vinegar on non-alcoholic fatty liver disease. Front. Pharmacol. 11, 585582. doi: 10.3389/fphar.2020.585582 33343352 PMC7747854

